# The Relationship between Nutrient Patterns and Bone Mineral Density in Postmenopausal Women

**DOI:** 10.3390/nu11061262

**Published:** 2019-06-03

**Authors:** Bolaji Lilian Ilesanmi-Oyelere, Louise Brough, Jane Coad, Nicole Roy, Marlena Cathorina Kruger

**Affiliations:** 1 School of Food and Advanced Technology, Massey University, Tennent Drive, Palmerston North 4442, New Zealand; l.brough@massey.ac.nz (L.B.); j.coad@massey.ac.nz (J.C.); 2 Food Nutrition & Health Team, AgResearch Grasslands, Palmerston North 4442, New Zealand; nicole.roy@agresearch.co.nz; 3 Riddet Institute, Massey University, Palmerston North 4442, New Zealand; m.c.kruger@massey.ac.nz; 4 High-Value Nutrition National Science Challenge, Auckland 1142, New Zealand; 5 School of Health Sciences, College of Health, Massey University, Palmerston North 4442, New Zealand

**Keywords:** nutrient pattern, dietary fat, dietary calcium, bone mineral density, osteoporosis

## Abstract

In women, the menopausal transition is characterized by acid-base imbalance, estrogen deficiency and rapid bone loss. Research into nutritional factors that influence bone health is therefore necessary. In this study, the relationship between nutrient patterns and nutrients important for bone health with bone mineral density (BMD) was explored. In this cross-sectional analysis, 101 participants aged between 54 and 81 years were eligible. Body composition and BMD analyses were performed using dual-energy X-ray absorptiometry (DXA). Nutrient data were extracted from a 3-day diet diary (3-DDD) using Foodworks 9 and metabolic equivalent (MET-minutes) was calculated from a self-reported New Zealand physical activity questionnaire (NZPAQ). Significant positive correlations were found between intakes of calcium (*p* = 0.003, r = 0.294), protein (*p* = 0.013, r = 0.246), riboflavin (*p* = 0.020, r = 0.232), niacin equivalent (*p* = 0.010, r = 0.256) and spine BMD. A nutrient pattern high in riboflavin, phosphorus and calcium was significantly positively correlated with spine (*p* < 0.05, r = 0.197) and femoral neck BMD (*p* < 0.05, r = 0.213), while the nutrient pattern high in vitamin E, α-tocopherol, β-carotene and omega 6 fatty acids was negatively correlated with hip (*p* < 0.05, r = −0.215) and trochanter BMD (*p* < 0.05, r = −0.251). These findings support the hypothesis that a nutrient pattern high in the intake of vitamin E, α-tocopherol and omega 6 fatty acids appears to be detrimental for bone health in postmenopausal women.

## 1. Introduction

Diet and hence nutrition are shown to be useful and modifiable tools for the management and possibly the prevention of metabolic bone disorders such as osteoporosis. This is especially true if good nutrition and a healthy balanced diet is introduced early in life, and followed through to adulthood [1,2]. In the past two decades, a growing body of literature has recognized the importance of nutrients in bone health [3,4] and dietary patterns [5]. However, few studies have investigated the impact of nutrient patterns on bone density [1,2]. Although investigating a single nutrient can be beneficial, research into a combination of nutrients in foods may help individuals with differing environment, culture, food habits and preferences putting into consideration the synergistic, additive and antagonistic effects of these nutrients when consumed together.

Many nutrients have been associated with increased BMD in many different populations alike. Nutrients readily available in fruits and vegetables (for example potassium and magnesium) have been linked with higher BMD in midlife women [6]. Studies have also linked an increase in calcium, phosphorus, magnesium and vitamin D intake with greater bone mineral density in postmenopausal women [7,8] as well as indicating an association between high protein intake and reduced incidence of fracture [9]. Results of the relationship between levels of homocysteine and B-vitamins, especially riboflavin and niacin, are few and inconsistent [10,11].

The Western diet, typically lacking alkali-forming metabolites provided by high fruit and vegetable intake, has been considered a factor for the higher prevalence of osteoporosis in the developed world [12]. This diet, also high in saturated fatty acids, may affect the bioavailability and bio-accessibility of calcium forming poorly digestible calcium-fatty acid soaps. Studies have also reported monounsaturated fatty acids (MUFAs) and polyunsaturated fatty acids (PUFAs) as negatively associated with BMD [12,13,14]. These findings were concluded to be a result of either hyperinsulinemia induced by a high fat diet leading to a negative calcium–magnesium balance or a reduced calcium absorption as a result of a high lipid diet due to the formation of calcium soaps [12].

The effects of the acid-base imbalance due to an increased consumption of a high fat diet might not be as pronounced in women who are still menstruating [12]. However, in postmenopausal women, an increase in steady state acid level with age [15] and estrogen deficiency, needs to be balanced with an increased intake of fruits and vegetables [6,12]. In postmenopausal women, it has been suggested that an increased acidity with age coupled with a high fat nutrient pattern may therefore be detrimental to bone mineral density [12,16]. Diet high in phosphorus and proteins and sulphur amino acids may also induce low-grade metabolic acidosis, thereby increasing dietary acidic load [17].

The influence of nutrient patterns on postmenopausal bone loss is not well understood as most research has focused on calcium, vitamin D and single nutrients but there is little work on nutrient patterns. In addition, older studies have used mainly food frequency questionnaires (FFQ) rather than diet records (which are considered to be the gold standard method) and many did not adjust for confounders such as body mass index (BMI) and physical activity. The aim of this current research was to investigate the relationship between nutrient patterns and also individual nutrients important for bone health with BMD in post-menopausal women.

## 2. Materials and Methods

### 2.1. Study Design

A total of 127 postmenopausal women aged between 54 and 81 years were recruited to participate in the cross-sectional study, ‘BugsnBones’, that took place in the Human Nutrition Research Unit of Massey University, Palmerston North campus from June to December, 2017. The sample size was calculated using G*Power software version 3.0.10 and eighty-eight participants were required for a 90% power and an alpha of 5% for a T-test, to test for the mean differences between two groups of osteoporotic and non-osteoporotic women based on the BMD values. A total of 110 women were evaluated.

Participants were recruited by advertisement on campus, the Whanganui Chronicle and by using a recruitment agency, Trial Facts (https://trialfacts.com/). Women at least 5 years post-menopause based on menstruation were included in the study. Exclusion criteria were presence of any systemic disease, food intolerances that affect the gastrointestinal tract, smokers and high intake of alcohol. Participants with significant weight loss or weight gain within the past year were excluded. Two participants were excluded post recruitment, one due to following a ketogenic diet and the other due to health conditions, while nine participants had missing diet records.

Evaluation of energy intake: basal metabolic rate using the Goldberg cut-off [18] revealed 3 (3%) over-reporters, 12 (10%) under-reporters and 101 (87%) plausible reporters. A total of 26 participants were therefore excluded from the study. All participants were free living and healthy. Written informed consent was obtained from participants before commencing data collection. The study was registered with the Australian New Zealand Clinical Trials Registry (ANZCTR) by the number ACTRN12617000802303. This study was also approved by Massey University Human Ethics Committee: Southern A, Application 17/17.

### 2.2. Anthropometric and Body Composition Measurements of the Participants

Fasting participants’ body weight was measured using the Detecto 437 eye-level weigh beam physician scale (USA) to the nearest 0.2 kg and standing height was measured using a wall-mounted stadiometer to the nearest 0.1 cm wearing light clothes and no shoes; BMI was calculated as weight divided by height squared (kg/m²). The waist to hip ratio was determined by measuring the waist and hip circumference to the nearest 0.1 cm using a non-stretchable tape. The waist to hip ratio was calculated as a marker of abdominal obesity.

Body composition measurements, fat mass, lean mass and fat percentage were measured and analyzed using the Hologic QDR series Discovery A, bone densitometer [Dual energy X-ray Absorptiometry (DXA)]. Bone mineral density was measured at the femoral neck (FN), lumbar spine (LS) [L1-L4], total hip and whole body. All measurements were conducted on the non-dominant body parts. The DXA machine was calibrated every morning for all the measurements and at the end of each day. The in vivo reproducibility of the coefficient of variation ranged between 0.34 and 0.70% for all measured sites. The reported BMD values were calculated as means of four measured values from L1–L4. Apex System Software version 4.5.3 (USA) was used for analyzing the DXA scans. Osteoporosis was defined as a T score ≤ 2·5 and osteopenia as T score between −1.0 and −2.5 according to the WHO criteria [18].

### 2.3. Dietary Intake Assessment

Participants’ dietary assessment of the food patterns was investigated with a 3-day diet diary (3-DDD) over non-consecutive days (including one weekend day). The 3-DDD was used to collect information on participants’ food and beverage intake utilizing household measures. The 3-DDD has been recommended to get information on mean food consumption with an advantage as the “golden standard” for dietary assessment [19]. Brand name of food products, recipes and method of food preparation were recorded. Food works 9 professional, Xyris software was used to analyze the participants’ diet data.

### 2.4. Physical Activity Questionnaire

Levels of physical activity were assessed using the New Zealand Physical Activity Questionnaire - short form (NZPAQ-SF) [20]. The NZPAQ has previously been validated by Boon et al. [21]. Physical activities were then quantified by METs -min/day which was calculated by using the scoring protocol of IPAQ for continuous score. MET values and formula for computation of MET-minutes was calculated and used as below [22].

- Walking MET-minutes/week at work = 3.3 × walking minutes × walking days at work.
- Moderate MET-minutes/week at work = 4.0 × moderate-intensity activity minutes × moderate intensity days at work.
- Vigorous MET-minutes/week at work = 8.0 × vigorous-intensity activity minutes × vigorous intensity days at work Total Work MET-minutes/week = sum of Walking + Moderate + Vigorous MET-minutes/week scores at work.
- Total PA MET-minutes/week = sum of walking + moderate + vigorous MET-minutes/week scores.

### 2.5. Nutrient Pattern Identification

Principal component analysis (PCA) was used to identify nutrient patterns using 36 nutrients collated from all measured nutrients. The analysis was performed for 101 study participants in order to reflect the nutrient patterns. Orthogonal (varimax) rotation was performed to reduce the correlations between factors and increase interpretability [2].

### 2.6. Data Analyses

IBM SPSS version 25 (IBM Company, Armonk, NY, USA) was used for all statistical analyses. The outcome variables used were BMD of the whole body and at skeletal sites. The values of all variables for the whole body and regional sites were presented as mean ± standard deviation. The independent variables consist of the nutrient intakes such dietary calcium, phosphorus, fat, protein and the nutrient patterns obtained from the dimension reduction of 36 nutrients. Variables were parametric and normally distributed. Correlation analyses of the whole body, regional sites BMD with the independent variables were performed to obtain the Pearson’s correlations. All *p*-values were reported significant at 0.05 or less.

## 3. Results

The mean age of the participants was 63 years (range = 54, 81years) and the mean (SD) spine BMD was 0.94 (0.15) g/cm^2^ (Table 1).

Three nutrient patterns were generated by PCA accounting for a total of 45.4% explained variance of the nutrient intakes (Table 2). NP1 was characterized by high levels of riboflavin, phosphorus, calcium and explained the variance of 24.1% while NP2 was characterized by high levels of dietary fats and fatty acids explaining the variance of 12.4% of the nutrient intake. In addition, NP3 characterized by high levels of vitamin A, fat and low levels of vitamin C and β−carotene explained the nutrient intake variance of 9.0%. Nutrient intakes across quartiles of each nutrient pattern are as shown in Table S1.

There was a trend of positive correlation between NP1 and all of the BMD sites even after adjusting with age, BMI and MET-minutes (Table 3). However, NP2 was mainly negatively correlated with the BMD sites. Nutrient pattern NP3 was also positively correlated with the BMD sites without adjustments but became negatively correlated when adjusted for BMI and MET-minutes. The confounding variables were adjusted for age because of the age range while BMI and MET-minutes was adjusted for due to the impact on BMD. The NP1 high in riboflavin, calcium and phosphorus was positively correlated with the spine BMD (*p* = 0.048, r = 0.197) and femoral neck BMD (*p* = 0.033, r = 0.213), while NP2 high in fats, vitamin E and fatty acids was significantly negatively correlated with total hip (*p* = 0.031, r = −0.215).

There was a trend of positive correlations across the nutrients high in the NP1 as well as the calcium/phosphorus ratio and niacin equivalent (Table 4). Interestingly, niacin equivalent and riboflavin had a strong positive effect on the BMD sites for the post-menopausal women even after adjustment for confounders. Calcium (*p* = 0.003, r = 0.294) and niacin equivalent (*p* = 0.010, r = 0.256) were weakly positively correlated with the spine BMD. These positive correlations were mainly significant for the spine and whole body BMD.

## 4. Discussion

This study indicates that a nutrient pattern higher in calcium, phosphorus, potassium, protein and the B vitamins (NP1) was positively correlated with whole body and skeletal sites BMD while a nutrient pattern higher in fats, vitamin E and fatty acids (NP2) was negatively correlated with BMD at all sites. Univariate analysis also showed significant positive correlations between intakes of calcium, protein, riboflavin, niacin equivalent and BMD of the spine. Calcium, riboflavin and niacin intakes were still significantly positively related with spine BMD even after adjusting for age, BMI and MET-minute. The calcium–phosphorus ratio was significantly positively associated with spine BMD.

Calcium, phosphorus, potassium and protein are well-known to have a beneficial effect on bone health [4,6,12,14], however, little research is available on the role of niacin and riboflavin on bone health, especially in older women. Although these B vitamins may not have a direct role in bone metabolism, their role in energy metabolism and as an essential cofactor for the enzyme ornithine decarboxylase plays a part in osteoblast NADPH concentrations for the vitamin K cycle [23]. Riboflavin, also referred to as vitamin B2, is a water-soluble vitamin important for cell growth and function, while niacin is important for keeping the bone stronger by retention of calcium [23,24]. Milk and dairy products have been reported as the greatest contribution to riboflavin intake in the Western diet [24].

Low or inadequate levels of vitamin B12, folate and vitamin B6 in the metabolism of homocysteine (Hcy) are known to cause hyper-homocysteinemia which is considered to be detrimental to bone health [25,26]. The mechanism linking increased homocysteine to increased risk of fracture has not yet been clarified, however, elevated levels of homocysteine have been shown to affect the cross-linking of collagen, thereby reducing bone strength [27,28].

The results of this study are similar to those of Shono et al. 1997 which found the effectiveness of riboflavin in maintaining a higher bone density in premenopausal Japanese women [29]. Furthermore, a similar study identified a significant positive correlation between niacin and BMD [30]. The NP1 was also high in vitamin B12 which is known as an important cofactor for osteoblast-related proteins such as osteocalcin and alkaline phosphatase [23]. It has therefore been suggested that it is important to set an appropriate recommended levels of B vitamins for optimal bone health [24].

The nutrient pattern was high in fats, vitamin E and alpha-tocopherol (NP2) and was mainly negatively correlated with BMD. Intake of fat and its influence on calcium absorption has also been reported with MUFAs and PUFAs, indicated as negatively associated with BMD [12,13,14]. Evidence as far back as the early 1900s suggests that fat interferes with calcium absorption through the formation of calcium soaps [31] which might be a reason for the negative relationship between fats and BMD. It has also been suggested that the possible mechanism for the negative associations between fat consumption and BMD may be as a result of high-fat-induced hyper-insulinema that may lead to a negative calcium–magnesium balance [12]. In addition, studies have reported the role of serum vitamin E and alpha-tocopherol in bone mass through the induction of osteoclast fusion in a mouse model in vivo and in vitro [32] and in a rat model [33]. This is, however, in contrast to the findings of Carvalho et al. 2013 [34], indicating that vitamin E does not prevent alveolar bone loss in another animal model. A large longitudinal study of 891 premenopausal women, conducted by Macdonald et al. 2004 [12], resulted in mixed outcomes; total vitamin E intake of dietary plus supplementation correlated positively with BMD, but was not significant while dietary vitamin E intake was significantly negatively correlated with BMD. The role and effect of vitamin E on bone is therefore not conclusive, as is supported by a review by Mohamed et al. [35].

Meanwhile, the nutrient pattern characterized by a high fat, protein, cholesterol and low in vitamin C (NP3) was positively correlated with BMD when performed in the crude state and when adjusted for age but negatively correlated when adjusted for BMI and Met-minutes. Accounting for the effect of age, BMI and MET-minutes, the NP3 was negatively associated with BMD except the whole body BMD and these were not significant. This is consistent with the findings by Melaku et al. 2017 [2] which found that an animal-sourced nutrient pattern high in fats, protein and cholesterol was positively associated with BMD crudely but negatively associated when adjusted for BMI and energy intake and likewise not significant. This finding could be a result of the effect of energy intake and physical activity which can be observed as confounders in this study.

The strength of this study includes the use of a 3DDD in the analysis of the data as well as the use of Goldberg cutoffs for identifying plausible reporters for the exclusion of under- and over-reporters of diet. Limitations of the study are its cross-sectional nature and the small sample size which may not allow generalization or cause and effect principle to be applied.

In summary, we found that a nutrient pattern high in fats, omega 6 fatty acids, alpha tocopherol and vitamin E was negatively correlated with BMD while a nutrient pattern high in calcium, phosphorus, riboflavin and niacin was positively correlated with BMD at all sites. The link between saturated fatty acids and a possible reduced calcium absorption due to calcium-fatty acid soaps formation needs more in-depth research. However, the limitations of this study are to be considered when interpreting the findings of this study. It should be noted that the high intakes mentioned are relative to the nutrient pattern in our cohort and intakes of protein must be regulated for the mild metabolic acidosis induced by overconsumption of sulfur-containing amino acids. Further research is therefore needed for longitudinal studies to support the nutrient pattern approaches, especially ones high in vitamin E, β-carotene and omega 6 fatty acids and its impact on bone mineral density and osteoporosis for both clinical and public health interventions.

## Figures and Tables

**Table 1:** General characteristics and nutrient intakes of 101 participants based on 3-DDD.

	Mean ± SD	Range	
Parameters	(*n* = 101)	Min	Max
Age (years)	62.9 ± 4.4	54	81
BMI (kg/m^2^)	26.3 ± 4.2	17.9	44.0
Waist-Hip ratio	0.8 ± 0.1	0.7	1.1
Spine (L1-L4) BMD (g/cm^2^)	0.9 ± 0.2	0.5	1.3
Spine (L1-L4) T-score	−1.0 ± 1.4	−4.6	2.6
Femoral neck BMD (g/cm^2^)	0.7 ± 0.1	0.5	1.0
Total hip BMD (g/cm^2^)	0.9 ± 0.1	0.6	1.2
Total hip T-score	−0.7 ± 1.0	−2.5	2.1
WB BMD (g/cm^2^)	1.1 ± 0.1	0.9	1.5
Energy Intake (kJ)	8109.8 ± 1727.5	4939.7	13,843.4
Protein (g)	85.6 ± 21.5	42.4	155.7
Total fat (g)	79.5 ± 22.4	33.2	128.9
Carbohydrate (g)	192.0 ± 64.4	61.8	394.6
Saturated fat (g)	30.5 ± 11.8	8.3	66.4
Polyunsaturated fat (g)	12.1 ± 6.0	3.4	48.8
Monounsaturated fat (g)	27.2 ± 8.7	11.0	51.3
Cholesterol (mg)	295.0 ± 146.1	18.2	753.5
Sugars (g)	96.1 ± 38.5	29.6	183.3
Starch (g)	95.8 ± 37.4	24.4	215.6
Dietary fibre (g)	27.2 ± 9.0	9.9	53.0
Vitamin C (mg)	118.2 ± 69.4	13.6	387.1
Vitamin D (µg)	6.3 ± 6.5	0.3	33.2
Vitamin E (mg)	10.6 ± 4.0	2.6	20.3
Vitamin B6 (mg)	2.4 ± 1.1	0.8	8.1
Vitamin B12 (µg)	4.2 ± 3.8	1.1	31.0
Vitamin K (µg)	19.9 ± 19.0	0.0	90.7
Vitamin A (µg)	1038.4 ± 860.5	291.1	8085.9
Riboflavin (mg)	2.1 ± 0.8	0.9	4.7
Alpha tocopherol (mg)	8.3 ± 3.2	2.4	17.2
Niacin equivalent (mg)	37.7 ± 10.5	16.7	66.6
Calcium (mg)	929.3 ± 358.9	336.7	2170.3
Sodium (mg)	2058.4 ± 816.7	616.2	5073.2
Phosphorus (mg)	1520.4 ± 383.1	810.2	2639.3
Iron (mg)	12.4 ± 5.3	4.7	48.2
Zinc (mg)	10.3 ± 3.2	4.9	20.6
Magnesium (mg)	372.2 ± 97.6	211.6	592.1
Potassium (mg)	3613.6 ± 933.2	1930.0	6448.8
Phytosterols (mg)	21.0 ± 42.6	0.0	218.2
Caffeine (mg)	251.0 ± 218.9	0.0	1834.1

BMI = body mass index; WB = whole body; BMD = bone mineral density.

**Table 2:** Factor loading matrix for the nutrient patterns of 101 participants.

	Nutrient Pattern * Factor Loadings		
	NP1	NP2	NP3
Riboflavin_mg	**0.829**		
Phosphorus_mg	**0.798**	0.106	**0.346**
Calcium_mg	**0.777**		
Sugars_g	**0.774**		
Potassium_mg	**0.765**	0.218	
Vitamin B6_mg	**0.759**		
Carbohydrate_g	**0.755**		0.110
Magnesium_mg	**0.700**	**0.450**	0.229
Thiamin_mg	**0.538**		−0.241
Sodium_mg	**0.529**		0.165
Iron_mg	**0.524**	0.259	0.164
Iodine_µg	**0.417**	0.130	
Niacin equivalent_mg	**0.392**		0.140
Vitamin B12_µg	**0.346**	0.101	
Retinol_µg			
Polyunsaturated fat_g		**0.811**	0.264
Vitamin E_mg	0.103	**0.807**	0.163
Alpha tocopherol_mg	0.114	**0.779**	0.116
Linoleic acid_g		**0.686**	0.178
Beta carotene_µg	0.291	**0.576**	**−0.410**
Alpha linolenic_ALA_g		**0.535**	0.138
Alpha_carotene_µg	**0.361**	**0.516**	**−0.350**
Eicosapentaenoic_EPA_g		**0.483**	
Docosahexaenoic_DHA_g	−0.168	**0.470**	
Vitamin A_µg	0.185	0.255	−0.171
Total fat_g	0.194	**0.373**	**0.776**
Monounsaturated fat_g		**0.499**	**0.708**
Oleic acid_g	0.114	**0.449**	**0.707**
Saturated fat_g	0.285	−0.113	**0.681**
Protein_g	**0.520**		**0.588**
Zinc_mg	**0.432**	0.125	**0.558**
Cholesterol_mg			**0.548**
Biotin_µg		0.121	**0.524**
Pantothenic acid_mg	0.227		**0.374**
Vitamin C_mg	0.279	0.202	**−0.339**
Erucic acid_g			0.133

NP, nutrient pattern; * Nutrient patterns were extracted by principal component analysis. Only factors loading >|0.1| are displayed. The bold text indicates a factor loading >|0.3|.

**Table 3:** Correlations of nutrient pattern scores and bone mineral density for 101 participants.

Bivariate Correlations				
Nutrient Pattern	Spine BMD	FN BMD	Hip BMD	WB BMD
NP1	0.197 *	0.213 *	0.103	0.194 *
NP2	−0.030	−0.175	−0.215 *	−0.115
NP3	0.112	0.103	0.172	0.168
Partial correlations adjusting for age				
NP1	0.198 *	0.186	0.073	0.177
NP2	−0.030	−0.193	−0.233 *	−0.124
NP3	0.112	0.107	0.178	0.171
Partial correlations adjusting for age and BMI				
NP1	0.261 **	0.240 *	0.133	0.199 *
NP2	0.062	−0.127	−0.158	−0.089
NP3	−0.061	−0.048	−0.009	0.107
Partial correlations adjusting for age, BMI and MET				
NP1	0.211 *	0.213 *	0.111	0.184
NP2	0.100	−0.111	−0.147	−0.079
NP3	−0.059	−0.046	−0.007	0.109

FN = femoral neck; Troch = trochanter; Inter = intertrochanteric; WB = whole body; * Correlation is significant at the 0.05 level (2-tailed); ** Correlation is significant at the 0.01 level (2-tailed).

**Table 4:** Correlations between selected nutrient intakes, calcium/phosphorus ratio and bone mineral density.

Bivariate Correlations				
	Spine BMD	FN BMD	Hip BMD	WB BMD
Calcium intake	0.294 **	0.206 *	0.148	0.239 *
Phosphorus intake	0.189	0.147	0.076	0.219 *
Ca/P ratio	0.257 *	0.160	0.154	0.165
Protein intake	0.246 *	0.182	0.178	0.241 *
Riboflavin intake	0.232 *	0.194	0.143	0.193
Niacin eq. intake	0.256 **	0.305 **	0.257 **	0.299 **
Partial correlations adjusting for age				
Calcium intake	0.295 **	0.187	0.127	0.226 *
Phosphorus intake	0.189	0.125	0.052	0.206 *
Ca/P ratio	0.257 *	0.146	0.140	0.155
Protein	0.246 *	0.181	0.177	0.239 *
Riboflavin intake	0.233 *	0.167	0.113	0.175
Niacin eq. intake	0.256 *	0.294 **	0.244*	0.290 **
Partial correlations adjusting for age and BMI				
Calcium intake	0.309 **	0.188	0.127	0.224 *
Phosphorus intake	0.184	0.114	0.030	0.200 *
Ca/P ratio	0.282 **	0.157	0.158	0.158
Protein	0.153	0.089	0.059	0.199 *
Riboflavin intake	0.300 **	0.219 *	0.179	0.198 *
Niacin eq. intake	0.211 *	0.256 *	0.194	0.268 **
Partial correlations adjusting for age, BMI and MET-minutes				
Calcium intake	0.251 *	0.152	0.099	0.207 *
Phosphorus intake	0.117	0.076	0.000	0.183
Ca/P ratio	0.259 *	0.140	0.145	0.148
Protein	0.131	0.075	0.048	0.192
Riboflavin intake	0.244 *	0.187	0.155	0.180
Niacin eq. intake	0.212 *	0.254 *	0.192	0.267 **

FN = femoral neck; Troch = trochanter; WB = whole body; * Correlation is significant at the 0.05 level (2-tailed); ** Correlation is significant at the 0.01 level (2-tailed).

## Supplementary Materials

The following are available online at https://www.mdpi.com/2072-6643/11/6/1262/s1, Table S1: Mean (SD) of selected nutrient intake across quartiles of nutrient pattern scores (*n* = 101).
