# Supplementary material for: The Relationship between Nutrient Patterns and Bone Mineral Density in Postmenopausal Women
Source: Nutrients. 2019 Jun 3;11(6):1262. doi: 10.3390/nu11061262 (PMC6628050; doi:10.3390/nu11061262)
Supplement: Supplementary file 1 [file nutrients-11-01262-s001.pdf]

Consumption patterns of selected nutrients across quartiles of nutrient patterns give a description of the nutrient patterns with Q1 being the lowest quartile and Q4 the highest quartile (Table S1)

Table S1: Mean (SD) of selected nutrient intake across quartiles of nutrient pattern scores (n=101)

	**NP1**					**NP2**				
**Nutrients**	**Q1**	**Q2**	**Q3**	**Q4**	***P*-value**	**Q1**	**Q2**	**Q3**	**Q4**	***P*-value**
Protein (g)	68.9 (18.0)	81.4 (16.7)	94.0 (13.6)	97.8 (24.3)	<0.001	83.9 (21.8)	82.4 (24.6)	89.7 (22.6)	86.5 (16.7)	0.651
Total fat (g)	70.3 (16.3)	77.7 (30.1)	87.1 (16.0)	82.6 (22.4)	0.049	71.3 (20.2)	71.3 (22.3)	85.8 (22.3)	89.8 (19.6)	0.002
Carbohydrate (g)	136.0 (35.3)	166.2 (47.7)	203.5 (40.5)	262.1 (53.1)	<0.001	195.1 (61.5)	190.8 (68.1)	194.9 (71.6)	187.4 (59.2)	0.971
Vitamin D (µg)	7.1 (9.2)	5.4 (4.6)	6.9 (7.0)	5.7 (3.8)	0.703	3.3 (2.1)	5.3 (3.9)	5.2 (3.6)	11.4 (10.1)	<0.001
Calcium (mg)	652.5 (193.5)	809.1 (184.6)	940.1 (248.2)	1315.0 (388.5)	<0.001	985.0 (404.1)	871.5 (321.7)	945.2 (404.8)	917.8 (307.3)	0.723
Magnesium (mg)	290.9 (54.5)	322.2 (67.3)	411.7 (76.7)	462.2 (80.2)	<0.001	321.5 (84.4)	335.5 (93.8)	389.1 (82.7)	444.2 (81.8)	<0.001
Phosphorus (mg)	1177.0 (224.2)	1345.4 (236.8)	1624.0 (180.2)	1931.1 (359.7)	<0.001	1504.0 (472.7)	1420.3 (382.1)	1569.0 (370.1)	1592.2 (283.2)	0.381
Potassium (mg)	2787.1 (456.2)	3216.8 (479.5)	3896.6 (770.7)	4542.8 (842.4)	<0.001	3359.0 (849.8)	3424.5 (859.8)	3748.4 (901.4)	3930.2 (1045.9)	0.095
Total omega 3	1.5 (0.9)	1.5 (1.2)	1.4 (0.9)	1.5 (1.2)	0.989	0.8 (0.4)	1.0 (0.5)	1.7 (1.3)	2.2 (1.1)	<0.001
Total omega 6	5.9 (2.6)	7.9 (6.4)	7.5 (3.5)	6.9 (2.9)	0.340	4.8 (1.9)	5.5 (2.2)	7.6 (3.0)	10.3 (5.9)	<0.001
Beta-carotene (µg)	2214.0 (1659.4)	1954.0 (1705.2)	2970.5 (1532.3)	3057.2 (2366.4)	0.089	1529.0 (783.7)	2107.6 (1388.8)	2520.0 (1649.7)	4073.6 (2364.8)	<0.001
Alpha-carotene (µg)	294.9 (336.0)	312.9 (661.1)	640.0 (506.7)	692.5 (716.8)	0.021	230.2 (259.8)	408.2 (435.6)	409.7 (584.0)	901.5 (777.1)	<0.001
Biotin (µg)	8.2 (5.8)	5.6 (3.4)	13.1 (13.3)	10.8 (15.1)	0.073	6.4 (5.6)	8.8 (9.5)	11.2 (14.4)	11.5 (11.8)	0.302
Vitamin C (mg)	82.1 (32.5)	125.9 (80.2)	135.3 (70.1)	128.7 (74.8)	0.024	104.1 (55.6)	102.4 (48.2)	141.6 (97.6)	125.2 (62.6)	0.139
Vitamin B12	3.5 (2.2)	3.5 (1.3)	3.6 (1.1)	6.4 (6.7)	0.012	3.5 (1.5)	3.9 (1.8)	4.9 (4.4)	4.7 (5.8)	0.490
Saturated fat	25.6 (8.1)	29.9 (13.5)	31.3 (7.6)	35.2 (15.0)	0.035	32.7 (13.6)	28.4 (11.1)	32.6 (12.8)	28.5 (9.4)	0.368
Cholesterol (mg)	296.2 (157.6)	299.4 (165.2)	313.3 (129.3)	270.3 (135.7)	0.771	266.6 (95.7)	292.8 (160.0)	355.3 (163.5)	265.2 (144.4)	0.098
Dietary fibre (g)	21.1 (4.5)	22.6 (5.3)	29.5 (8.4)	35.4 (8.9)	<0.001	23.8 (8.7)	23.7 (6.0)	27.5 (7.4)	33.9 (9.8)	<0.001
Energy kJ	6529.2 (813.1)	7548.5 (1626.7)	8818.1 (945.5)	9515.0 (1632.7)	<0.001	7797.6 (1733.7)	7791.4 (1973.9)	8414.5 (1713.8)	8448.4 (1416.0)	0.333
MET-minutes	310.2 (359.4)	252.1 (216.9)	292.6 (245.9)	800.5 (1433.2)	0.036	438.8 (699.5)	533.9 (1233.6)	333.7 (553.1)	339.4 (307.0)	0.771

One-way ANOVA was used to test the difference across the quartiles.

Table S1: Mean (SD) of selected nutrient intake across quartiles of nutrient pattern scores (n=101) contd.

	NP3				
**Nutrients**	**Q1**	**Q2**	**Q3**	**Q4**	***P*-value**
Protein (g)	69.2 (16.5)	84.5 (18.4)	89.9 (18.3)	98.9 (21.9)	<0.001
Total fat (g)	59.5 (17.3)	71.4 (13.8)	82.0 (15.8)	105.3 (13.0)	<0.001
Carbohydrate (g)	182.4 (62.3)	194.9 (64.8)	179.8 (58.2)	210.9 (70.8)	0.305
Vitamin D (µg)	4.8 (6.2)	6.2 (4.5)	8.7 (9.6)	5.4 (3.7)	0.163
Calcium (mg)	887.4 (419.8)	971.3(318.4)	886.4 (321.9)	970.4 (379.2)	0.716
Magnesium (mg)	343.8 (100.5)	376.5 (107.7)	355.7 (76.1)	412.7 (94.0)	0.064
Phosphorus (mg)	1333.2 (410.8)	1532.9 (382.0)	1526.4 (294.1)	1688.6 (371.8)	0.011
Potassium (mg)	3570.5 (1031.1)	3692.8 (964.3)	3555.3 (826.0)	3632.8 (949.4)	0.952
Total omega 3	1.3 (1.2)	1.2 (0.6)	1.6 (1.0)	1.6 (1.3)	0.452
Total omega 6	6.6 (6.4)	6.6 (3.0)	6.8 (2.4)	8.2 (3.8)	0.441
Beta-carotene (µg)	3512.5 (2584.7)	2614.4 (1959.0)	2405.6 (1244.5)	1677.4 (801.7)	0.005
Alpha-carotene (µg)	779.1 (864.1)	524.8 (563.9)	407.1 (393.7)	233.8 (271.9)	0.009
Biotin (µg)	5.2 (3.2)	7.0 (4.7)	8.2 (6.4)	17.5 (17.9)	<0.001
Vitamin C (mg)	151.4 (88.3)	115.7 (71.9)	103.4 (49.6)	102.3 (52.6)	0.039
Vitamin B12	4.8 (6.9)	3.9 (1.7)	3.6 (1.4)	4.7 (2.5)	0.613
Saturated fat	20.4 (5.7)	27.9 (8.3)	32.4 (10.1)	41.5 (11.6)	<0.001
Cholesterol (mg)	194.6 (93.1)	245.4 (102.9)	353.7 (133.2)	388.1 (160.5)	<0.001
Dietary fibre (g)	28.9 (9.9)	27.7 (10.7)	25.1 (7.9)	26.9 (7.1)	0.509
Energy kJ	6973.7 (1425.0)	7833.4 (1565.3)	8125.4 (1363.3)	9517.7 (1581.1)	<0.001
MET-minutes	608.0 (1341.6)	365.1 (629.7)	326.4 (340.3)	353.0 (347.8)	0.549

One-way ANOVA was used to test the difference across the quartiles.
